# Aβ promotes CD38 expression in senescent microglia in Alzheimer’s disease

**DOI:** 10.1186/s40659-022-00379-1

**Published:** 2022-03-03

**Authors:** Yiran Hu, Yan Huang, Sanli Xing, Chuan Chen, Dingzhu Shen, Jiulin Chen

**Affiliations:** 1 Shanghai Geriatric Institute of Chinese Medicine, 365C South Xiangyang Road, Shanghai, 200031 China; 2 Shanghai University of Traditional Chinese Medicine, 1200 Cailun Road, Pudong New Area, Shanghai, 200031 China

**Keywords:** Microglia, Senescence, Energy dysmetabolism, Neuroinflammation, NAD +, CD38

## Abstract

**Background:**

In Alzheimer’s disease (AD), the neuroinflammatory response mediated by the activation of senescent microglia is closely related to energy dysmetabolism. However, the mechanism underlying the interaction between the energy metabolism of aging microglia and neuroinflammation remains unclear.

**Methods:**

We used biochemical methods, enzyme-linked immunosorbent assay (ELISA), immunofluorescence, and western blot to determine the effects and mechanism of CD38 knockdown on energy metabolism and neuroinflammation in Aβ1-40 injured BV2 cells. Using AD model mice, we detected CD38 enzyme activity, energy metabolism factors (ATP, NAD +, and NAD + /NADH), and neuroinflammatory factors (IL-1β, IL-6, and TNF-α) following the addition of CD38 inhibitor. Using a combination of biochemical analysis and behavioral testing, we analyzed the effects of the CD38 inhibitor on energy metabolism disorder, the neuroinflammatory response, and the cognition of AD mice.

**Results:**

Following Aβ1-40 injury, SA-β-Gal positive cells and senescence-related proteins P16 and P21 increased in BV2 cells, while energy-related molecules (ATP, NAD +, and NAD + /NADH) and mitochondrial function (mitochondrial ROS and MMP) decreased. Further studies showed that CD38 knockdown could improve Aβ1-40-induced BV2 cells energy dysmetabolism and reduce the levels of IL-1β, IL-6, and TNF-α. In vivo results showed an increase in senile plaque deposition and microglial activation in the hippocampus and cortex of 34-week-old APP/PS1 mice. Following treatment with the CD38 inhibitor, senile plaque deposition decreased, the number of Iba1 + BV2 cells increased, the energy metabolism disorder was improved, the proinflammatory cytokines were reduced, and the spatial learning ability was improved.

**Conclusions:**

Our results confirm that senescent microglia appeared in the brain of 34-week-old APP/PS1 mice, and that Aβ1-40 can induce senescence of BV2 cells. The expression of CD38 increases in senescent BV2 cells, resulting in energy metabolism disorder. Therefore, reducing CD38 expression can effectively improve energy metabolism disorder and reduce proinflammatory cytokines. Following intervention with the CD38 inhibitor in APP/PS1 mice, the energy metabolism disorder was improved in the hippocampus and cortex, the level of proinflammatory cytokines was reduced, and cognitive impairment was improved.

## Background

Alzheimer’s disease (AD) is a neurodegenerative disease that is characterized by a gradual decline in memory and cognitive ability [1]. Amyloid plaques formed by extracellular β deposition, and neurofilament tangles formed by intracellular tau protein hyperphosphorylation are the main pathological features of AD [2]. The cause of AD is complex and remains not fully understood. The pathogenesis of AD is complex, and there is no effective treatment for AD at present. The clinical treatment drugs for AD are mainly to improve symptoms and alleviate progression, such as cholinesterase inhibitors [3, 4]. The aging population has dramatically increased worldwide, and the proportion of adults aged 60 years or older is expected to reach 22% by 2050. China’s population aging situation is also grim, with > 200 million elderly individuals and the highest rate of population aging and the highest number of elderly people worldwide [5, 6]. This aging population makes AD a major medical and social problem, posing a huge challenge to social and economic development in China. The incidence rate of AD increases gradually during the aging process; indeed, aging is an important risk factor for neurodegenerative diseases, including AD [7].

As immune cells in the central nervous system, microglia play a key role in brain development and immune defense and can also clear Aβ deposition [8]. Under normal physiological conditions, microglia constantly observe the microenvironment to maintain brain homeostasis. Under abnormal stress, microglia can rapidly expand their cell processes, migrate to the injured site, remove harmful substances, and induce an immune response by releasing inflammatory factors, such as Interleukin-1β (IL-1β), Interleukin-6 (IL-6) and tumor necrosis factor-α (TNF-α) [9]. Aβ production and clearance exist in a dynamic balance, and its concentration in the brain remains stable, partly dependent on microglia phagocytizing and removing excess Aβ oligomer in the brain [10]. However, the degradation of Aβ aggregates after phagocytosis is quite slow, so the effectiveness of microglia scavenging Aβ is a key question. Autophagy is considered to be one of the important processes of Aβ fiber degradation. Studies have shown that microglia phagocytose fibrils rather than oligomers, but the latter are the main Aβ form that induces the production of inflammatory cytokines and inhibits the phagocytosis of fibrillary Aβ [11]. With the progress of AD, microglia undergo an age-associated decrease in microglial ability to interact with Aβ. With aging, activated microglia cannot effectively clear Aβ, and the release of inflammatory cytokines are increased [12]. Thus, Aβ oligomers can activate microglia to produce numerous inflammatory cytokines, ultimately leading to cognitive decline [13]. Many studies have shown that the AD mouse model exhibits a microglia-mediated neuroinflammatory response; therefore, inhibiting the inflammatory activation of microglia is a potential strategy for treating AD [14].

Studies have found that maintaining the stability of the energy metabolism of microglia plays an important role in maintaining their normal function [15]. Under normal circumstances, microglia produce vast quantities of adenosine triphosphate (ATP) through oxidative phosphorylation, while the rates of oxidative phosphorylation and glycolysis are decreased in aging microglia [16]. Moreover, in aging, the metabolic model of microglia changes from oxidative phosphorylation to glycolysis, decreasing the rate of ATP production and leading to energy metabolism disorder [17]. The rate of ATP production is regulated by nicotinamide adenine dinucleotide (NAD +). NAD + receives high-energy electrons from glycolysis and the tricarboxylic acid cycle (TCA), and finally sends the electrons to complex I of the electron transfer chain (ETC) to drive oxidative phosphorylation, which is the main source of ATP [18]. In the process of aging, the NAD + level significantly decreases, which also decreases the level of ATP. It has been found that activation of the NAD + degrading enzyme CD38 in aging microglia is the main reason for the decrease in NAD + observed during aging [19]. CD38 is widely expressed in immune cells, and it directly degrades NAD + and NAD + precursors, such as nicotinamide mononucleotide (NMN) and nicotinamide riboside (NR), to decrease the NAD + level. Additionally, the CD38 level and activity increase with aging [20].

In this study, we first observed senescent cells and Iba1 + microglia in the hippocampus and cortex of 34-week-old APP/PS1 and C57/BL mice. In vitro, we used Aβ1-40 to injure BV2 cells and observe changes related to senescence in BV2 cells. We next analyzed the energy metabolism, including energy metabolism molecules (NAD +, ATP) and mitochondrial function (ROS, MMP). Many studies have shown that CD38 overactivation is one of the main reasons for the decrease in NAD + observed during aging. Inhibition of CD38 overexpression can significantly improve the NAD + level. Subsequently, knockdown and overexpression of CD38 were performed to analyze whether the energy dysmetabolism in BV2 cells could be improved and to further explore the effect on the neuroinflammatory response, including the levels of pro-inflammatory factors (IL-1β, IL-6, TNF-α). Finally, we treated APP/PS1 mice with a CD38 inhibitor to analyze the effect of inhibiting CD38 expression in Iba1 + microglia on energy metabolism disorder and neuroinflammation in the hippocampus and cortex. We also sought to further analyze the effect of inhibiting CD38 expression in Iba1 + microglia on plaque deposition and behavioral damage in the elderly.

## Results

### Cell senescence in the hippocampus and cortex of APP/PS1 mice

**Fig. 1:**
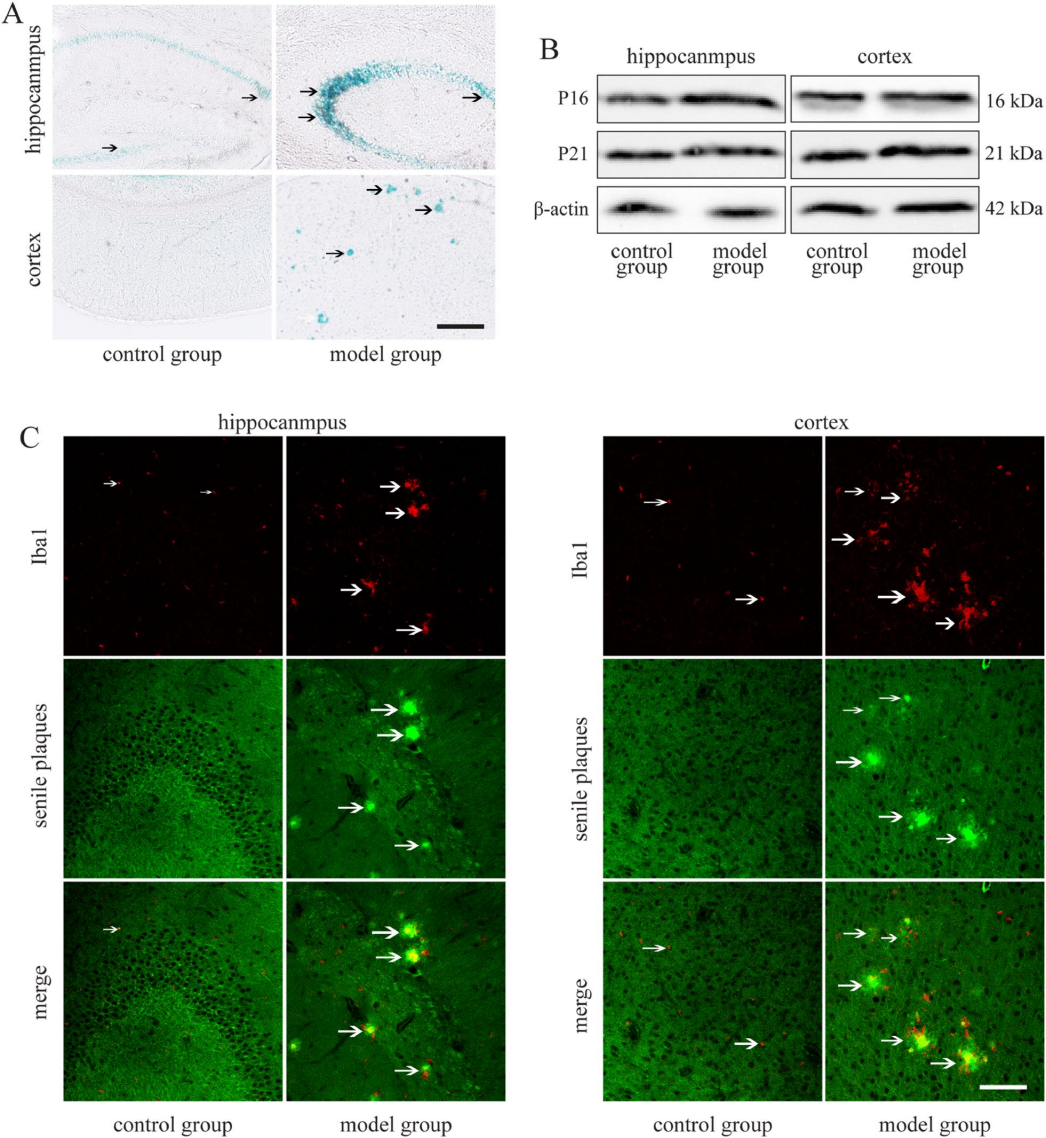
Cell senescence in the hippocampus and cortex of APP/PS1 mice. Scale bar: 50 μm. **A** SA-β-Gal staining in the hippocampus and cortex of APP/PS1 and C57/BL6J mice. **B** P16 and P21 protein expression levels in the hippocampus and cortex of APP/PS1 and C57/BL6J mice. **C** Representative images of Iba1 + and senile plaques immunolabeling in the hippocampus and cortex of APP/PS1 and C57/BL6J mice

Interestingly, the accumulation of senescent cells is an important cause of brain aging. Senescent cells are accompanied by changes in metabolism, which are closely related to functional changes [21]. As SA-β-Gal is an important marker of cell senescence [22], we used the Senescence β-Galactosidase staining kit to detect the cellular senescence of the hippocampus and cortex in each group of mice. The results showed that the SA-β-Gal positive stained area in the hippocampus and cortex of the model group was significantly higher than that of the control group (Fig. 1A).

An abnormal cell cycle is a key pathological feature of cell senescence; senescent cells show permanent cell cycle arrest, which is mainly regulated by P16INK4a protein and P53-P21-RB protein [23]. Western blot results showed that expression of P16 and P21 protein in the hippocampus and cortex of the model group was increased compared to the control group (Fig. 1B). These results indicate that the number of senescent cells in the hippocampus and cortex of 34-week-old APP/PS1 mice increased.

In the pathogenesis of AD, insufficient degradation of Aβ deposition is the driving force of AD brain pathology; however, whether activated microglia near the Aβ plaque accelerate or delay the progression of AD is controversial [24]. We labeled activated microglia marker Iba1 + around the senile plaque deposition. The results showed that senile plaque deposition increased in the hippocampus and cortex of APP/PS1 mice. Moreover, while Iba1 + microglia appeared around the senile plaque, the Iba1 + microglia had shorter processes and round or rod-shaped cell morphology (Fig. 1C).

### Aβ1-40 induced energy metabolism disorder and mitochondrial dysfunction in BV2 cells

**Fig. 2:**
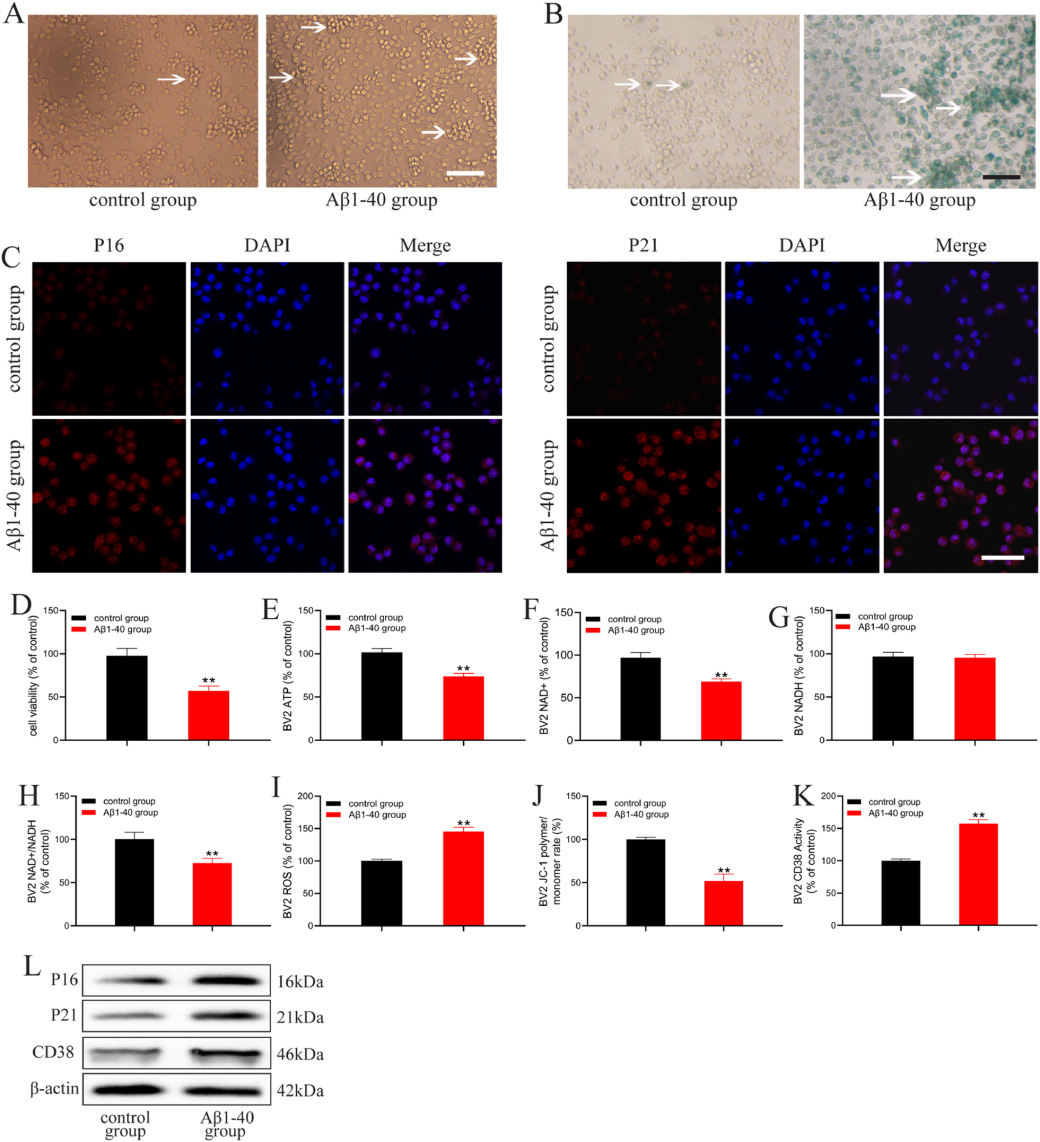
Aβ1-40 induced energy metabolism disorder and mitochondrial dysfunction in BV2 cells. Scale bar: 50 μm. **A** Morphological changes of BV2 cells treated with Aβ1-40 (1 μM). **B** SA-β-Gal positive stained area. **C** Representative images of BV2 cells immunolabeled for P16 (red) and P21 (red). Nuclear staining (DAPI) is shown in blue. **D** Cell viability of BV2 cells **E** ATP level of BV2 cells. **F** NAD + level of BV2 cells. **G** NADH level of BV2 cells. **H** NAD + /NADH ratio of BV2 cells. **I** ROS level of BV2 cells. **J** MMP level of BV2 cells. **K** CD38 enzymatic activity of BV2 cells. **L** P16, P21, and CD38 protein expression level of BV2 cells. All data are expressed as the mean ± standard error. **P < 0.01 vs. control group

As activated microglia surround the deposition of senile plaques, we sought to explore whether the activation of microglia is caused by insoluble Aβ. We used 1 μM Aβ1-40 to intervene in BV2 cells. The results showed that the BV2 cells were round with a clear profile. After treatment with Aβ1-40, BV2 cells were processed, and a few cells had an unclear outline (Fig. 2A). After 24 h of Aβ1-40 intervention, the viability of BV2 cells decreased significantly (Fig. 2D). We also evaluated the effect of Aβ1-40 on the senescence of BV2 cells. The results showed that the SA-β-Gal positive cells induced by Aβ1-40 were significantly increased (Fig. 2B), and the expression of P16 and P21 protein was increased (Fig. 2L), suggesting that Aβ1-40 may induce BV2 cells senescence. As aging microglia decrease the rate of ATP production, we analyzed the ATP levels to determine whether Aβ1-40 could injure the energy metabolism in BV2 cells. The results showed that the ATP level decreased after Aβ1-40 treatment (Fig. 2E). NAD + is a metabolic molecule related to energy metabolism, which can regulate ATP production. We also measured the levels of NAD +, NADH, and NAD + /NADH in Aβ1-40 injured BV2 cells. The results showed that Aβ1-40 significantly reduced the NAD + and NAD + /NADH ratio of BV2 cells (Fig. 2F, H), leading to energy metabolism disorder. We further examined the mitochondrial function of BV2 cells and found that after Aβ1-40 treatment, mitochondrial ROS was increased and MMP was decreased in BV2 cells (Fig. 2I, J), suggesting the occurrence of mitochondrial dysfunction. In summary, Aβ1-40-injured BV2 cells induced mitochondrial function decline and energy metabolism disorder.

CD38 not only degrades NAD + but also degrades NAD + precursors such as NMN and NR [25]. Overactivation of the NAD + degrading enzyme CD38 is thought to be one of the main reasons for the rapid decrease in NAD + observed during aging. To determine whether CD38 is involved in Aβ1-40-induced energy dysmetabolism in BV2 cells, the enzyme activity and protein expression of CD38 was detected in Aβ1-40 injured BV2 cells. Biochemical tests showed that CD38 enzymatic activity was significantly increased in Aβ1-40 injured BV2 cells, as was CD38 protein expression (Fig. 2K, L).

### CD38 knockdown improves energy dysmetabolism and reduces the expression of inflammatory factors in Aβ1-40 injured BV2 cells

**Fig. 3:**
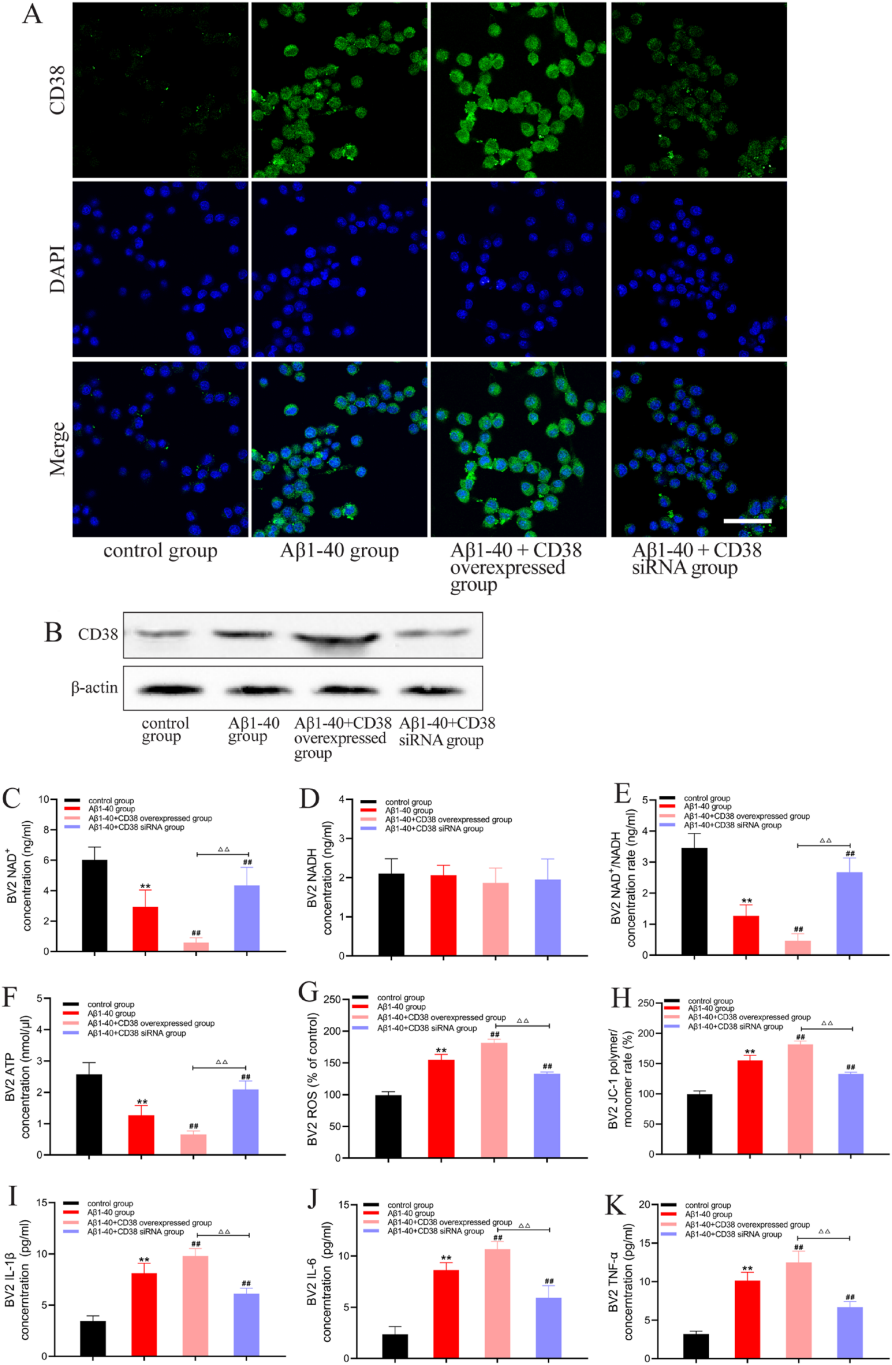
CD38 knockout improves energy dysmetabolism and reduces the expression of inflammatory factors in Aβ1-40 injured BV2 cells. Scale bar: 50 μm. **A** Representative image of BV2 microglial cells exposed to Aβ1-40 (1 μM), and overexpressed or knockdown CD38 immunolabeled for CD38 (green). Nuclear staining (DAPI) is shown in blue. **B** CD38 protein expression level of BV2 cells. **C** NAD + level of BV2 cells. **D** NADH level of BV2 cells. **E** NAD + /NADH ratio of BV2 cells. **F** ATP level of BV2 cells. **G** ROS level of BV2 cells. **H** MMP level of BV2 cells. **I** IL-1β level of BV2 cells. **J** IL-6 level of BV2 cells. **K** TNF-α level of BV2 cells. All data are expressed as the mean ± standard error. **P < 0.01 vs. control group; ^##^P < 0.01 vs. Aβ1–40 group; ^△△^P < 0.01

As Aβ1-40 induced CD38 expression in BV2 cells, we further clarified the role of CD38 in energy metabolism and neuroinflammation by knocking down and over-expressing CD38 (Fig. 3A, B). The results showed that knockdown of CD38 increased the NAD + level and NAD + /NADH ratio in Aβ1-40 injured BV2 cells, whereas CD38 overexpression had the opposite effect (Fig. 3C, E). We also examined the ATP levels and mitochondrial function and found that CD38 knockdown increased the ATP level in Aβ1-40 injured BV2 cells (Fig. 3F), increased MMP (Fig. 3G), and decreased mitochondrial ROS (Fig. 3H). These results suggest that CD38 knockdown improves energy metabolism disorder and mitochondrial function, while CD38 overexpression aggravates energy dysmetabolism and mitochondrial dysfunction in BV2 cells injured by Aβ1-40.

The energy metabolism of microglia plays an important role in regulating their function, and CD38 knockdown can alleviate energy metabolism disorder in Aβ1-40 injured BV2 cells. We further investigated whether CD38 knockdown could reduce the neuroinflammatory response in Aβ1-40 injured BV2 cells. The results showed that after CD38 knockdown, the levels of IL-1β, IL-6, and TNF-α inflammatory cytokines were decreased in Aβ1-40 injured BV2 cells, while overexpression of CD38 increased the levels of these cytokines (Fig. 3I–K). Together, these results suggest that decreasing CD38 can improve energy metabolism and can further alleviate the neuroinflammatory response in BV2 cells.

### Inhibition of CD38 improves energy metabolism disorder in APP/PS1 mice and reduces the neuroinflammatory response

**Fig. 4:**
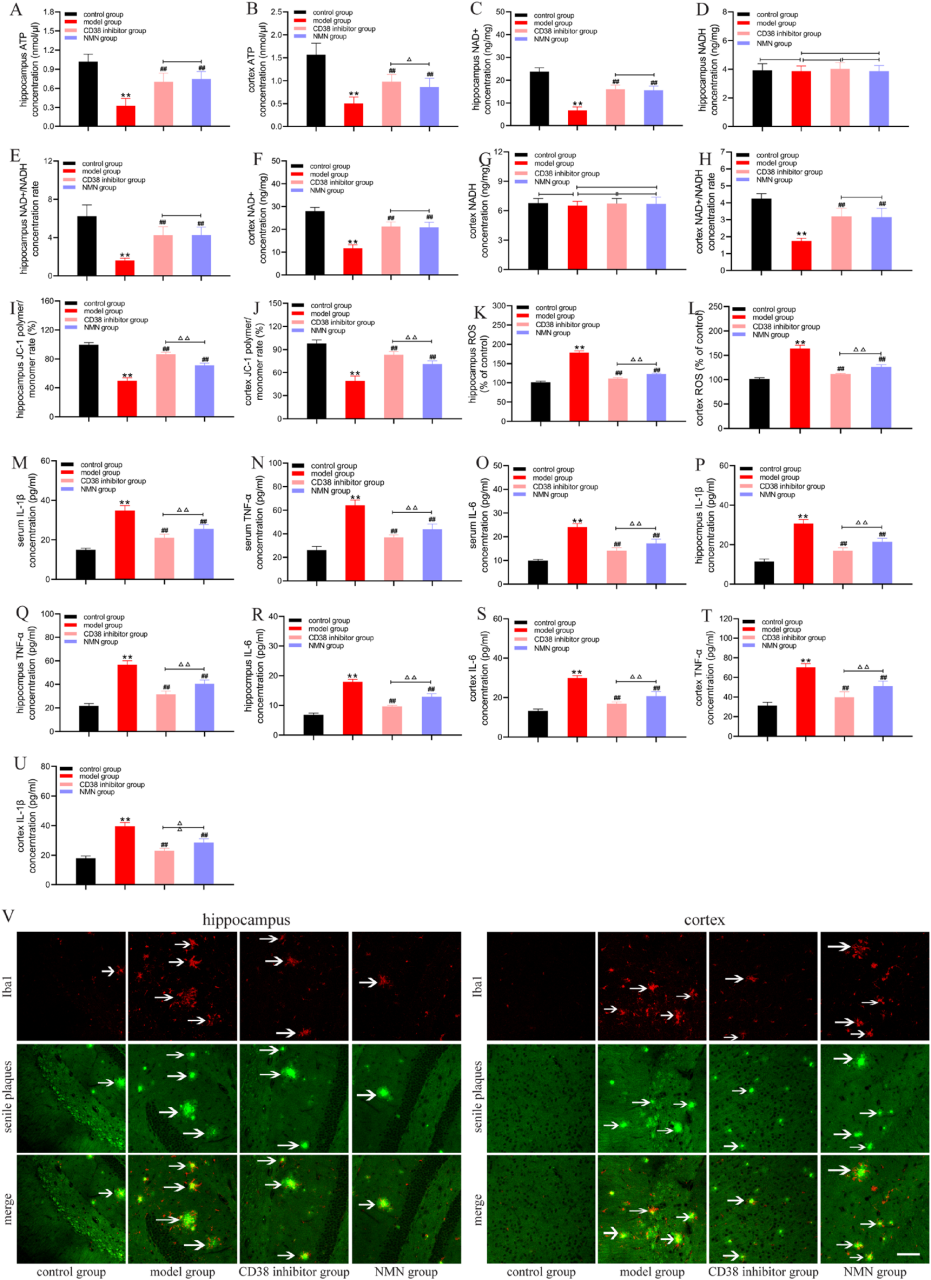
Inhibition of CD38 expression improves energy metabolism disorder in APP/PS1 mice and reduces the neuroinflammatory response. Scale bar: 50 μm. **A** ATP level of the hippocampus. **B** ATP level of the cortex. **C** NAD + level of the hippocampus. **D** NADH level of the hippocampus. **E** NAD + /NADH ratio of the hippocampus. **F** NAD + level of the cortex. **G** NADH level of the cortex. **H** NAD + /NADH ratio of the cortex. **I** MMP of the hippocampus. **J** MMP of the cortex. **K** ROS level of the hippocampus. **L** ROS level of the cortex. **M** IL-1β level of the serum. **N** TNF-α level of the serum. **O** IL-6 level of the serum. **P** IL-1β level of the hippocampus. **Q** TNF-α level of the hippocampus. **R** IL-6 level of the hippocampus. **S** IL-6 level of the cortex. **T** TNF-α level of the cortex. **U** Representative images of Iba1 + and senile plaque immunolabeling in the hippocampus and cortex. *P < 0.05 and **P < 0.01 vs. control group; ^#^P < 0.05 and ^##^P < 0.01 vs. model group; ^△^P < 0.05 and ^△△^P < 0.01

Knockdown of CD38 can improve energy metabolism disorder and reduce the neuroinflammatory response in Aβ1-40 injured BV2 cells. We studied the effect of the CD38 inhibitor in an AD mouse model. The results showed that the ATP levels in the hippocampus and cortex of the model group were lower than those of the control group (Fig. 4A, B). Compared to the control group, the NAD + level and NAD + /NADH ratio in the hippocampus and cortex of the model group were decreased, while the addition of CD38 inhibitor led to the recovery of the NAD + level, NAD + /NADH, and improved the energy metabolism disorder (Fig. 4C–H). We also found that the mitochondrial ROS increased and MMP decreased in the hippocampus and cortex of 34-week-old APP/PS1 mice, respectively, while the CD38 inhibitor reduced the mitochondrial ROS and increased the MMP (Fig. 4I–L). These results suggest that the expression of CD38 increases in AD mice, resulting in energy metabolism disorder and mitochondrial dysfunction, while inhibition of CD38 expression improves energy metabolism disorder and mitochondrial dysfunction.

It has been found that restoring the energy metabolism of microglia can regulate the activation and function of microglia. Therefore, we further detected the activation status and neuroinflammatory response of microglia in the hippocampus and cortex of APP/PS1 mice. The results showed that the levels of IL-1β, IL-6, and TNF-α in the hippocampus and cortex of the model group were significantly higher than those of the control group, while the release of inflammatory factors decreased after CD38 inhibitor intervention (Fig. 4M–U). These results suggest that intervention with CD38 inhibitor in 34-week-old APP/PS1 mice not only improves energy dysmetabolism but also decreases neuroinflammatory cytokines.

Finally, we observed the senile plaque deposition and labeled activated microglia with Iba1 +. The results showed that intervention with the CD38 inhibitor decreased the number of senile plaques in the hippocampus and cortex of APP/PS1 mice compared to the model group, and the accumulation of Iba1 + microglia decreased. These findings may indicate that the dysfunction of Iba1 + microglia was improved and that the coverage and phagocytic ability of Iba1 + microglia over senile plaques are enhanced. These results revealed that in the hippocampus and cortex of 34-week-old APP/PS1 mice, microglia are stimulated by senile plaque deposition, the function of the Iba1 + microglia in scavenging and phagocytizing senile plaque deposition is decreased, and vast quantities of neuroinflammatory cytokines are secreted. Inhibition of CD38 expression improved the energy metabolism of the Iba1 + microglia, reduced the release of neuroinflammatory cytokines, and played a neuroprotective role (Fig. 4V).

### Inhibition of CD38 expression improves the cognitive learning ability of APP/PS1 mice

**Fig. 5:**
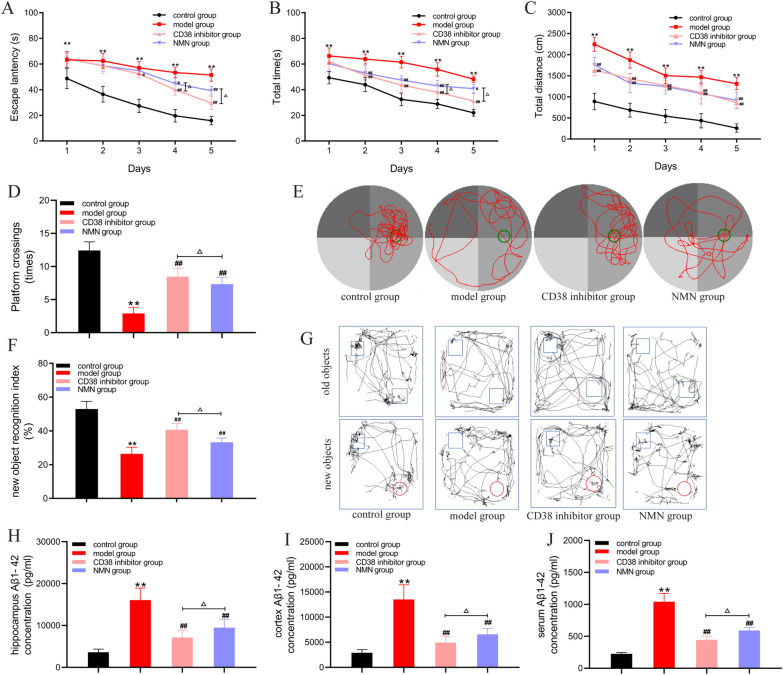
Inhibition of CD38 expression improves the cognitive learning ability of APP/PS1 mice. **A** Escape latency. **B** Total time. **C** Total distance. **D** Platform crossings. **E** Swimming trajectory. **F** New object recognition index. **G** New object recognition trajectory. **H** Aβ1-42 concentration of the hippocampus. **I** Aβ1-42 concentration of the cortex. **J** Aβ1-42 concentration of the serum. *P < 0.05 and **P < 0.01 vs. control group; ^#^P < 0.05 and ^##^P < 0.01 vs. model group; ^△^P < 0.05 and ^△△^P < 0.01

We further observed whether inhibition of CD38 can improve learning and memory in AD mice. We found that the escape latency of APP/PS1 mice was significantly longer than that of the control group during the first 5 days of learning training, the total distance was also significantly longer, and the latency and total distance of APP/PS1 mice were significantly shorter after treatment with the CD38 inhibitor (Fig. 5A–C). In the space exploration experiment on the 6th day, the number of mice that explored the original platform decreased significantly in the APP/PS1 group after removing the platform, and the number of APP/PS1 mice crossing the original platform increased after intervention with the CD38 inhibitor (Fig. 5D, E). The new object recognition experiment was used to detect the ability of mice to distinguish new and old objects. The results showed that the new object recognition index of APP/PS1 mice decreased, indicating impaired ability to distinguish new objects. After intervention with the CD38 inhibitor, the new object recognition index was enhanced, demonstrating that the impaired cognitive ability of APP/PS1 mice can be improved by treatment with a CD38 inhibitor (Fig. 5F–G).

We found that the CD38 inhibitor reduced senile plaque deposition in the hippocampus and cortex of APP/PS1 mice. In AD, amyloid precursor protein (APP) is decomposed into Aβ fragments by β-proteases and γ-protease, mainly including the Aβ1-40 fragment and Aβ1-42 fragment, where the Aβ1-42 fragment is more neurotoxic. We detected the Aβ1-42 levels in the hippocampus, cortex, and serum of mice, all of which were significantly higher in APP/PS1 mice compared to the control group. After intervention with the CD38 inhibitor, the Aβ1-42 content in the hippocampus, cortex, and serum of APP/PS1 mice decreased (Fig. 4H–J). These results suggest that CD38 inhibitors not only reduce senile plaque deposition but also reduce the Aβ1-42 level.

## Discussion

Aging is an important risk factor for neurodegenerative diseases. During aging, the function and structure of the brain change, and the cognitive ability of the brain decreases, which is manifested as a decline in learning and memory ability, decision-making speed, sensory perception, and motor coordination [26]. The learning behavior regulated by the hippocampus and cortex gradually degenerate with senescence [27]. Moreover, it has been reported that senile plaque deposition begins to increase significantly in AD mice aged 8–9 months [28]. So, we used 30-week-old APP/PS1 mice in the experiment, to give 4 weeks intervention, and the mice were 34 weeks old when experiments was carried out. Consistent with this, our results showed that 34-week-old APP/PS1 mice had impaired learning and cognition. The SA-β-Gal positive stained area and the expression of senescence proteins P16 and P21 increased in the hippocampus and cortex, suggesting that the number of senescence cells was increased in the hippocampus and cortex of 34-week-old APP/PS1 mice.

As innate immune cells in the brain, microglia account for approximately 10% of all cells in the nervous system [29]. Microglial over-activation is a hallmark of the aging brain and coincides with age-related neurodegeneration and cognitive decline [30]. The activation of microglia is closely related to the deposition of senile plaques. Sensing of Aβ peptide by microglia results in the elimination of Aβ peptide and other harmful substances. Microglia activated by Aβ plaques can clear cell debris and recruit immune cells to modulate a local immune response to Aβ [31]. The action of microglia and senile plaque deposition is a double-edged sword. Although microglia are useful in detecting the brain environment, the continuous production of senile plaque deposition and the chronic interaction with microglia can reduce the ability of microglia to clear senile plaque deposition and lead to phagocytic dysfunction, exacerbating the deterioration of AD [32]. Age-associated microglial dysfunction leads to cellular senescence and can profoundly alter the response to chronic inflammation [33]. However, our knowledge of microglia aging and the factors that regulate age-related microglial dysfunction remain limited [34]. Senescent microglia accumulate in the process of brain aging, and play different roles in different stages of AD. In the early stage of AD, microglia are mainly affected by Aβ, can be activated, present as an M2 phenotype, decompose, and phagocytize Aβ. However, with the aggravation of AD, the function of microglia decreases, and the inflammatory factors increase, showing an M1 phenotype. In AD, senile plaque deposition can activate M1 microglia, reduce the phagocytosis and clearance of senile plaque deposits, and release neurotoxic substances, such as TNF-α, IL-1β, IL-6, chemokines, and reactive oxygen and nitrogen species, leading to an increased neuroinflammatory response and neurodegeneration [35, 36]. Studies have found that inhibition of M1 microglia activation can alleviate cognitive dysfunction. Our results show that 34-week-old APP/PS1 mice have increased deposition of senile plaques in the hippocampus and cortex, as well as increased Iba1 + microglia around senile plaques, suggesting the impaired ability of Iba1 + microglia to phagocytose Aβ, and increased levels of neuroinflammatory cytokines TNF-α, IL-6, and IL-1β in the hippocampus and cortex. At this point, microglia cells present an M1 state. Therefore, senile plaques deposition may activate Iba1 + microglia, which not only reduces the ability of phagocytosis and clearance of amyloid plaques but also intensifies the neuroinflammatory response in microglia.

Activated microglia should produce ATP as an energy supply, but ATP is decreased in senescent microglia [37]. NAD + plays an important role in ATP production, and is an important coenzyme factor to maintain cell energy metabolism (such as in glycolysis, the tricarboxylic acid cycle, and electron transport chain) [38]. Intracellular NAD + homeostasis depends on the balance between NAD + synthetase and NAD + degrading enzyme, while CD38 overactivation may be important for the rapid decrease in NAD + in aging microglia [39]. The expression of CD38 in Aβ1-40 injured BV2 cells and the hippocampus and cortex in APP/PS1 mice increased, while a decrease in CD38 expression could restore the level of NAD + , increase the level of ATP, and improve energy metabolism and mitochondrial dysfunction.

Studies have found that microglia can clear Aβ plaques, reduce the neuroinflammatory response and mitochondrial dysfunction, and maintain energy metabolism homeostasis; together, these processes help to clear Aβ plaques and reduce the microglia-mediated neuroinflammatory response [40]. In resting microglia, NAD + homeostasis can maintain anti-inflammatory homeostasis and phagocytosis. However, in aging microglia, there is a complex relationship between metabolism and immune function. Decreased NAD + levels associated with aging may lead to a pro-inflammatory state of microglia and decreased clearance of Aβ [41]. Our results showed that after CD38 expression was reduced and the NAD + level of aging microglia was improved, the senile plaque deposition in the hippocampus and cortex of 34-week-old APP/PS1 mice decreased, the number of Iba1 + microglia around senile plaque decreased, and the inflammatory cytokine level decreased. Moreover, CD38 overexpression in Aβ1-40 injured BV2 cells led to a significant decrease in NAD + and an increase in inflammatory cytokines, suggesting a close relationship between CD38-mediated energy metabolism disorder and neuroinflammation in aging microglia.

## Conclusion

In this study, we found that microglia senescence occurred, and there was an increase in Iba1 + microglia around senile plaque deposition in 34-week-old APP/PS1 mice. Energy metabolism disorder caused by CD38 overactivation occurs in senescent Iba1 + microglia, which reduces their ability to phagocytose and clear senile plaque deposits. We also found that CD38 inhibitor intervention can restore the level of NAD + in the hippocampus and cortex of APP/PS1 mice, alleviate the disorder of energy metabolism, enhance the effect of Iba1 + microglia in clearing senile plaque deposits, reduce the neuroinflammatory response, and improve the learning and memory ability of APP/PS1 mice. The microglia energy metabolism mediated by CD38 is crucial to the function of microglia in AD. Therefore, improving the energy metabolism and neuroinflammatory response of microglia by targeting CD38 may be a strategy for treating AD.

## Materials and methods

### Reagents

Mouse microglia BV2 cells were purchased from the Cell Bank of Chinese Academy of Sciences; Dulbecco’s modified Eagle medium (12100046), fetal bovine serum (10099141C), and penicillin/streptomycin (10378016) were purchased from Gibco; Aβ protein fragment 1–40 (A1075) was purchased from Sigma-Aldrich; Lipofectamine 2000 Transfection Reagent (ab51243) was purchased from Abcam; CD38 siRNA (AM160089) was purchased from Invitrogen; CD38 CRISPR activating plasmid (sc-419548), CD38 antibody (SC374650), and M-IgGκ BP-HRP (sc-516102) were purchased from Santa Cruz Biotechnology; anti-rabbit IgG HRP-linked antibody (5490S) and β-actin mouse monoclonal antibody (3700) were purchased from Cell Signaling Technology; P21 rabbit antibody (AF5252), P16 rabbit antibody (AF1069), Iba1 rabbit antibody (AF7143), Cell Proliferation and Cytotoxicity Assay Kit (C0009), Enhanced ATP Assay Kit (S0027), Reactive Oxygen Species Assay Kit (S0033S), Mitochondrial Membrane Potential Assay Kit (C2006), and Mouse amyloid beta peptide 1–42 ELISA Kit (MU30114) were purchased from Bioswamp; Mouse TNF-α ELISA Kit (PT512), Mouse IL-6 ELISA Kit (PT312), Mouse IL-1β ELISA Kit (PT512), Senescence β-Galactosidase Staining Kit (C0602), Alexa Fluor 488 goat anti-rabbit IgG (a0423), DAPI-staining solution (C1006), phosphate buffered saline (C0221A) and bovine serum albumin (ST025) were purchased from Shanghai Beyotime Biotechnology; and NAD/NADH Quantitation Colorimetric Kit (K337) was purchased from Biovision.

### Animal grouping

Fourteen 30-week-old C57BL/6J mice were used as controls, and forty-two 30-week-old APP/PS1 mice were selected as AD model mice. The AD model mice were randomly divided into the model group, the CD38 inhibitor group, and the NMN group. The CD38 inhibitor and NMN were injected intraperitoneally once a day for 4 week, with 0.2 ml each time. The CD38 inhibitor was dissolved in 10% DMSO + 90% distilled water. NMN was also dissolved in distilled water to a concentration of 0.093 mg/ml, and CD38 inhibitor and NMN solution were intraperitoneally injected at 30 weeks old (0.0186 mg/d) once a day for 4 weeks. All of the administration operations and tissue extractions were authorized by the Ethics Committee of Shanghai University of Traditional Chinese Medicine, according to the animal protection law and experimental method (No. PZSHUTCM191025004).

### Senescence β-Galactosidase staining

According to the instructions of the Senescence β-Galactosidase Staining Kit, we first prepared the cells and brain slices in a 24-well plate. Next, 400 μl phosphate buffered saline (PBS) was added to the plate and washed three times (× 5 min per wash). Subsequently, 400 μl β-galactosidase staining fix solution was added, discarded after 20 min, and further washed three times with PBS (× 10 min per wash). After washing, 400 μl staining solution was added. The plate was wrapped to prevent evaporation before placing in a 37 °C incubator overnight. Following incubation, the slides were observed under an ordinary optical microscope (BX63, Olympus Corporation, Japan).

### Thioflavin-S staining

Three brain slices from each group were placed in 24-well plates, 400 μl PBS was added, and the slices were washed three times (× 5 min per wash). Next, 400 μl 1% Thioflavine-S solution was added and incubated in the dark at room temperature with shaking for 10 min. After staining, the Thioflavin-S dye solution was discarded, and 600 μl distilled water was added to each well before washing three times (× 5 min per wash). The brain slices were sealed and observed under a fluorescence microscope (BX51, Olympus Corporation, Japan).

### Western blot

Protein samples were heated for 5 min at 95 °C, separated by SDS polyacrylamide gel electrophoresis, and then transferred to polyvinylidene fluoride (PVDF) membrane. The PVDF membrane was sealed with 10% bovine serum albumin (BSA) for 1 h, and the primary antibody was added at a 1:2000 dilution before incubating overnight at 4 °C. After incubation, the membranes were washed with TBST (10 min × three washes). Next, the corresponding secondary antibody was added at a 1:1000 dilution before incubating at 37 °C for 1 h, followed by further washing with TBST (three times × 15 min per wash). ImageJ (V1.8.0.112) was used to analyze the protein bands to integrate the absorbance (IA = mean OD × Area). The relative level of the target protein was normalized to β-actin (target Protein IA/β-actin IA).

### Immunofluorescence

After the intervention, the BV2 cells were fixed with 4% paraformaldehyde for 15 min. Then BV2 cells and brain slices were washed with PBS for 10 min and then penetrated with 1% Triton for 10 min. A 10% BSA-blocking solution was sealed for 1 h, and the primary antibody was added at a ratio of 1:1000, incubated at 37 °C for 1 h and 4 °C overnight. After washing with PBS (three times × 10 min), the fluorescent secondary antibody was diluted with PBS at a ratio of 1:500, and DAPI was added at a ratio of 1:500. After incubation at 37 °C for 1 h, the cells were washed further with PBS.

### Aβ oligomer (AβO) preparation

The Aβ1-40 fragment was prepared as described above [42]. Briefly, 0.5 mg of Aβ1-40 lyophilized powder was placed at room temperature for 0.5 h. Then, PBS containing 0.05% sodium dodecyl sulfate was used to dissolve Aβ1-40, which was then mixed at a concentration of 100 µmol/L. Subsequently, Aβ1-40 was incubated at 37 °C for 7 days and stored at 4 °C for further use in the following experiments.

### Cell culture and treatments

BV2 cells were cultured in DMEM containing 10% FBS and 1% penicillin/streptomycin in 6-well plates. The cells were passaged when the fusion rate reached 80–90%. The cells were divided into the control, Aβ1-40, Aβ1-40 + CD38 siRNA, and Aβ1-40 + CD38 overexpressed groups. The control group was cultured with DMEM without any treatment for 24 h; the Aβ1–40 group was cultured with DMEM containing 1 μM Aβ1-40 for 24 h; and the Aβ1-40 + CD38 siRNA group was cultured with DMEM containing 1 μM Aβ1-40 for 24 h. Then, CD38 siRNA was transfected into the BV2 cells. The Aβ1-40 + CD38 overexpressed group was treated with 1 µM Aβ1-40 for 24 h, then the CD38 CRISPR plasmid was transfected into BV2 cells.

### MTT assay

According to the instructions of the MTT Cell Proliferation and Cytotoxicity Assay Kit, each well of cells was supplemented with 100 µl MTT solution (0.5 mg/ml) and incubated at 37 °C for 4 h. The supernatant was discarded, and 100 µl dimethyl sulphoxide was added to fully dissolve the crystals. Subsequently, absorbances were measured at a wavelength of 570 nm using a PowerWave XS microplate reader (BioTek Instruments, Inc., Winooski, VT, USA).

### ATP measurements

An enhanced ATP assay kit was used to determine the ATP levels as previously described. Briefly, the sample and lysate were mixed, and, after full pyrolysis, was centrifuged at 12,000 g for 5 min at 4 °C. Next, the supernatant was collected, and 100 μl of supernatant was added to each well of a 96-well plate and placed at room temperature for 5 min, before adding 20 μl working solution for ATP detection. The relative light unit (RLU) value was measured at 450 nm with a PowerWave XS microplate reader.

### NAD + /nicotinamide adenine dinucleotide hydride (NADH) measurements

First, 400 µl NAD_total_ was extracted according to the kit instructions. Subsequently, 200 µl NAD_total_ samples were transferred and heated at 60 °C for 30 min, and then centrifuged at 12,000 g for 30 s at 4 °C. The supernatants were labeled as NADH_samples_ for further assays. The standard curve was configured, 40 µl samples were loaded into the wells, and NAD + /NADH extraction buffer was added to a total volume of 50 µl. Finally, 100 µl enzyme-reaction mix and 10 µl NADH developer were added. After 4 h, the absorbance at 450 nm wavelength was measured using a PowerWave XS microplate reader.

### Mitochondrial extraction and mitochondrial membrane potential (MMP) measurements

Mitochondria were extracted from cells and brain according to the instructions of the Mitochondrial Isolate Kit. The MMP was measured using the mitochondrial membrane potential assay kit with JC-1. The prepared JC-1 staining-working solution was diluted five times, and 0.9 ml JC-1 staining-working solution was added to 0.1 ml purified mitochondria with total protein of 10–100 µg. The change in MMP was detected by the change in fluorescence color using a PowerWave XS (BioTek Instruments, Inc.) microplate reader at 490 nm excitation wavelength and 530 nm emission wavelength.

### Detection of reactive oxygen species (ROS)

Changes in intracellular ROS levels were determined by measuring the fluorescent dichlorofluorescein (DCF). DCFH-DA was diluted with serum-free DMEM (1:1000) and incubated at 37 °C for 20 min in the dark. Then, the cells were washed in serum-free DMEM to remove redundant DCFH-DA. The fluorescence intensity of DCF was measured with 488/525 nm excitation/emission wavelengths via a PowerWave XS microplate reader.

### Detection of CD38 enzyme activity

To detect CD38 enzyme activity, sucrose buffer was added to an appropriate amount of cells. Next, ultrasonic treatment was conducted on ice, with 30–50 W power and 20 kHz frequency, 5 s each time for a total of three times. After ultrasonic treatment, the sample was centrifuged at 4 °C, 13.8 *g* for 10 min, and the supernatant was collected. When preparing the reaction mixture, 1 ml of total reaction mixture contained 5 μl 10 mM ε- NAD and 40 μl 10 mM NGD. The reaction mixture was added to an opaque 96-well plate, and 100 μl sucrose buffer was added to each well (including the blank well). The values were read at an excitation wavelength of 300 nm and an emission wavelength of 410 nm using a power wave XS plate reader.

### Detection of neuroinflammatory factors (IL-1β, IL-6, TNF-α)

According to the instructions of the kit, six concentrations of standard (2000, 1000, 500, 250, 125, and 62.5 pg/ml) and samples were prepared and added to the corresponding wells at 100 μl/well. After incubation at room temperature for 2 h and subsequent washing, 300 μl cleaning solution was added to each well, followed by 100 μl horseradish peroxidase-labeled streptavidin. After incubation for 1 h, the plates were washed and 100 μl TMB solution was added to each well before incubating at room temperature in the dark for 20 min. After incubation, 50 μl termination solution was added to each well. After mixing, the absorbance was measured at 450 nm with a PowerWave XS microplate reader.

### Morris water maze experiment

The Morris water maze test was used to test the spatial learning and memory ability of mice in each group. The water pool was divided into four quadrants (I, II, III, and IV), and the platform was placed 1 cm below the water surface. In the quiet state, the mice were put into the water from the center of the four quadrants. The time taken for the mice to find the platform within 70 s was calculated. If the mice failed to find the platform, the time was calculated as 70 s. The experiment lasted for 5 days; on the sixth day, the space exploration experiment was conducted, and the number of times that the mice crossed the original platform in 70 s without placing the platform was calculated.

### New object recognition experiment

We further tested the cognitive ability of the mice using a new object recognition experiment. At the beginning of the training, two objects (A and B) were placed on the test box, and the mice were put into the field. After 10 min, the contact between the two objects was recorded. Then, object B was replaced by Object C. The mice were put into the field in the same way. After 5 min, the exploration of Object C by the mice was recorded. The new object recognition index (NOI) was calculated as the time of exploring new objects/(time of exploring new objects + time of exploring on-site objects) × 100%.

### ELISA for the determination of Aβ

The Aβ1-42 concentration was measured using the Mouse amyloid-beta peptide 1–42 ELISA Kit using a standard curve to determine the Aβ1-42 level. A 40 μl sample and 10 μl biotin labeled anti-Aβ1-42 antibody was added to the sample well. Finally, 50 μl enzyme-labeling reagent was added and incubated at 37 °C for 30 min. Following incubation, the wells were washed and 50 μl of reagents A and B were added separately. The absorbance values of the microplate were read at 450 nm using a PowerWave XS microplate reader.

### Statistical analysis

Data analysis was performed using GraphPad Prism 7 (GraphPad Software, Inc., La Jolla, CA, USA). The data are expressed as mean ± standard error. Normality and variance homogeneity were determined using the Shapiro–Wilk and Levene’s tests, respectively. Variance analyses were completely randomized. The Bonferroni *t*-test and Wilcoxon rank-sum test were used for pairwise comparisons. Meanwhile, the Kruskal–Wallis test was used for the analysis of nonparametric data. P-values < 0.05 were considered to indicate a statistically significant difference.

## Data Availability

The data that support the findings of this study are available from the corresponding author upon reasonable request.

## Abbreviations

AD: Alzheimer’s disease
ATP: Adenosine triphosphate
NAD +: Nicotinamide adenine dinucleotide
NADH: Nicotinamide adenine dinucleotide hydride
TCA: Tricarboxylic acid cycle
ETC: Electron transfer chain
NMN: Nicotinamide mononucleotide
NR: Nicotinamide riboside
ELISA: Enzyme-linked immunosorbent assay
PBS: Phosphate-buffered saline
AβO: Aβ oligomer
SA-β-Gal: Senescence-associated β-galactosidase
IL-1β: Interleukin-1β
IL-6: Interleukin-6
TNF-α: Tumor necrosis factor-α
MMP: Mitochondrial membrane potential
ROS: Reactive oxygen species
